# Monitoring data to explore particle size distribution and elemental composition in a stormwater outlet from a German urban catchment

**DOI:** 10.1016/j.dib.2026.112581

**Published:** 2026-02-13

**Authors:** Karen L. Rojas-Gómez, Jakob Benisch, Björn Helm, Dietrich Borchardt, Peter Krebs

**Affiliations:** a Institute of Urban Water Management, Dresden University of Technology, 01069 Dresden, Germany; b Department of Aquatic Ecosystem Analysis and Management, Helmholtz-Centre for Environmental Research (UFZ), 39114 Magdeburg, Germany; c Institute of Hydrobiology, Dresden University of Technology, 01069 Dresden, Germany

**Keywords:** Discharge, Elements, Fine sediments, Metals, Particle-bound pollutants, Separated drainage network, Turbidity, Urban drainage

## Abstract

High-resolution data and continuous monitoring of water quality parameters enable a more accurate characterisation of stormwater pollutants dynamics. This article presents a unique dataset combining real-time online monitoring of turbidity and discharge data with event-based, size-fractionated chemical characterisation of stormwater. Turbidity and discharge were measured with a high temporal resolution at the stormwater outlet of a small urban catchment in Dresden, Germany. Additionally, for selected rainfall-runoff events, the following data were produced: total suspended solids concentrations and their particle-size distribution (<63 µm: fine particles; >63 µm: coarse fraction), elemental composition, and organic content. The online monitoring data covers the period from January 2018 to August 2022, whereas the sampled data were collected from September 2018 to 2021. Turbidity serves as a proxy for particles, organic, and elemental composition of stormwater. Therefore, our dataset is suitable for exploring flush dynamics, particle transport patterns, particle-bound pollutants, as well as for developing and validating particle transport formulations in urban drainage models. This will enable a more effective identification of stormwater treatment and management strategies to address different pollutant flushes, support regulatory decision-making, and minimise the impact of stormwater discharges on receiving water bodies. Hence, intended users of this dataset include, but are not limited to, the urban drainage/urban hydrology/stormwater research community and practitioners, students, decision-makers, policymakers, urban planners, engineers, and other stakeholders interested in water-related issues at the city or urban catchment scale.

Specifications Table

**Table:** 

Subject	Earth & Environmental Sciences
Specific subject area	Monitoring suspended solids, elemental composition and particle-bound pollutants in stormwater
Type of data	Raw data Table (.csv format)
Data collection	The online monitoring station was equipped with online sensors for turbidity, flow rate and automatic sampling (MAXX GmbH) during storm events. A wedge sensor recorded velocity by the cross-correlation method and water level by a pressure cell (POA sensor, NIVUS) to measure discharge. A solitax-sc turbidity sensor (Hach) measured turbidity with a time resolution of 1 min. Stormwater samples were collected during storm events using the automatic sampler and were analysed for: total suspended solids (TSS), their fine fraction (<63 µm, FSS), coarse fraction (>63 µm, CSS), organic matter content using the Loss on Ignition (LOI %) method and elemental composition using an inductively coupled plasma optical emission spectrometry (ICP-OES, PerkinElmer).
Data source location	Stormwater outlet from an urban catchment in Dresden, Germany (51.004014° N, 13.844630° E). This monitoring station is part of the urban observatory managed by the working group on Urban Hydrology of the Institute for Urban Water Management - TU Dresden, Germany
Data accessibility	Repository name: OPARA, Open Access Repository and Archive for Research Data of Saxon Universities Digital Object Identifier: https://doi.org/10.25532/OPARA-1052 Direct URL to data: https://opara.zih.tu-dresden.de/handle/123456789/1915
Related research article	Rojas-Gómez, K. L., Benisch, J., Helm, B., Borchardt, D., & Krebs, P. (2026). Identifying flush and transport patterns driving particle export and elemental composition of stormwater from a German urban catchment. Journal of Hydrology: Regional Studies, 64, 103,196. https://doi.org/10.1016/j.ejrh.2026.103196 [1]

## 1 Value of the Data

• Our dataset comprises high-resolution turbidity and discharge monitoring data continuously measured during four years under real-world conditions at the outlet of a stormwater sewer network. The samples taken during rainfall events provide data on fine suspended solids, total suspended solids, organic, and elemental composition. The combination of long-term continuous proxy data (i.e., turbidity and discharge) with event-based, size-fractionated chemical characterisation (e.g., elemental composition) highlights the unique aspect of this dataset. This paired structure is particularly valuable for developing and calibrating models that link hydrology to water quality at the urban catchment scale.
• This dataset may be used in a range of urban drainage studies focusing on stormwater and/or urban drainage networks (UDN). This may include: i) analysing the transport and export of particles and particle-bound pollutants from stormwater outlets (SWO); ii) defining design criteria for stormwater treatment facilities, and iii) supporting regulatory decision-making regarding the export of fine sediments in stormwater and particle-bound pollutants such as metals.
• Acknowledging the importance of collaborative research and data sharing to design and manage urban drainage systems, researchers and practitioners can use this data to conduct further analysis on flush dynamics in stormwater outlets and to validate new approaches to simulate sediment and/or particle-bound pollutant transport in a stormwater network.
• This dataset can be used to explore the potential of real-time stormwater control using turbidity as an easy-to-measure proxy parameter related to particle-bound pollutants such as heavy metals. This information can be used to predict concentrations of contaminants during rainfall events at a high temporal resolution and to calculate mass balances. Hence, our dataset can be used as a benchmark for data-driven operation and planning of UDNs, supporting, for example, the assessment of their performance and evaluating management alternatives.
• Considerable monitoring, sampling, analytical efforts and resources have been used to generate this dataset to characterise a typical stormwater outlet from a city in Germany. This dataset may be used in future studies to avoid duplication of this effort, especially for researchers or practitioners focusing on this region (e.g., the German Association for Water, Wastewater and Waste Management – DWA).
• Having a dataset with a clear description of discharge, turbidity, particle fractionation, and elemental composition of stormwater in an urban catchment might allow other researchers to explore the possible impact of SWO on the receiving water bodies using water quality and hydrodynamic simulations.

## 2 Background

This dataset supports an original research article [1,2]. The data was collected to provide a further understanding of the flush dynamics and transport patterns of particles and the elemental composition of stormwater. The collected continuous turbidity and discharge data through online monitoring were essential to capture the stormwater sediment dynamics of a SWO more accurately. Previous studies [3,4] have demonstrated that continuous turbidity measurements can serve as a high-resolution monitoring strategy for total suspended solids and particle-bound pollutants, as suspended particles in water generate light scattering. Hence, by combining data from online monitoring and from grab samples during rainfall events, we described the export and transport patterns of suspended solids and associated pollutants, such as metals, at the SWO [1]. This allowed us to understand the dynamic transport mechanisms of fine sediments and relevant elements attached to particles, considering complex runoff and discharge processes. Since data availability in the urban drainage research and practice communities remains a significant challenge [5], our dataset [2] integrates real-time monitoring with event-sampling data to further support evidence-based urban water management and innovation.

## 3 Data Description

The dataset [2] includes continuous discharge (Q) and turbidity measurements from January 2018 to August 2022 with a one-minute time resolution. The TSS, FSS, CSS, organic content and bulk elemental composition of stormwater samples taken during rainfall events includes: i) 265 grab samples with TSS, FSS and CSS data during September 2018 - 2021; ii) 157 grab samples with TSS, FSS, CSS and organic content of each fraction and iii) concentration of elements (Al, B, Ba, Ca, Co, Cr, Cu, Fe, K, Li, Mg, Mn, Na, Ni, P, Pb, Sr and Zn) in 32 stormwater samples from four storm events. The dataset is divided into three sub-datasets in a .csv format, with the following file names and headlines:

**Main dataset:** Q_Turbidity_MS5.csv

**Date:** time of the measurement in YEAR-MONTH-DAY HOUR:MINUTE:SECOND (YYYY-MM-DD hh:mm:ss) with a time zone equal to “UTC”

**Q_lps:** discharge data in L/s

**FNU_SolitaxSteel:** turbidity data in FNU

**Sub-dataset 1:** SuspendedSolids_MS5.csv

**Date:** time of the measurement in YEAR-MONTH-DAY HOUR:MINUTE:SECOND (YYYY-MM-DD hh:mm:ss) with a time zone equal to “UTC”

**TSS_mgL:** total suspended sediment data from grab samples in mg/L

**FSS_mgL:** fine fraction of the total suspended sediment data (< 63 µm) from grab samples in mg/L

**CSS_mgL:** coarse fraction of the total suspended sediment data (> 63 µm) from grab samples in mg/L

**oFSS_mgL:** organic content of FSS from grab samples in mg/L

**oCSS_mgL:** organic content of CSS from grab samples in mg/L

**Sub-dataset 2:** Elements_MS5.csv

**Date:** time of the measurement in YEAR-MONTH-DAY HOUR:MINUTE:SECOND (YYYY-MM-DD hh:mm:ss) with a time zone equal to “UTC”

Additional columns show the bulk element concentration of: Al, B, Ba, Ca, Co, Cr, Cu, Fe, K, Li, Mg, Na, Mn, Ni, P, Pb, Sr, and Zn. All these concentrations are measured in the bulk sample and reported in mg/L. NA values are reported concentrations below the detection limit (DoL).

## 4 Experimental Design, Materials and Methods

**Fig. 1:**
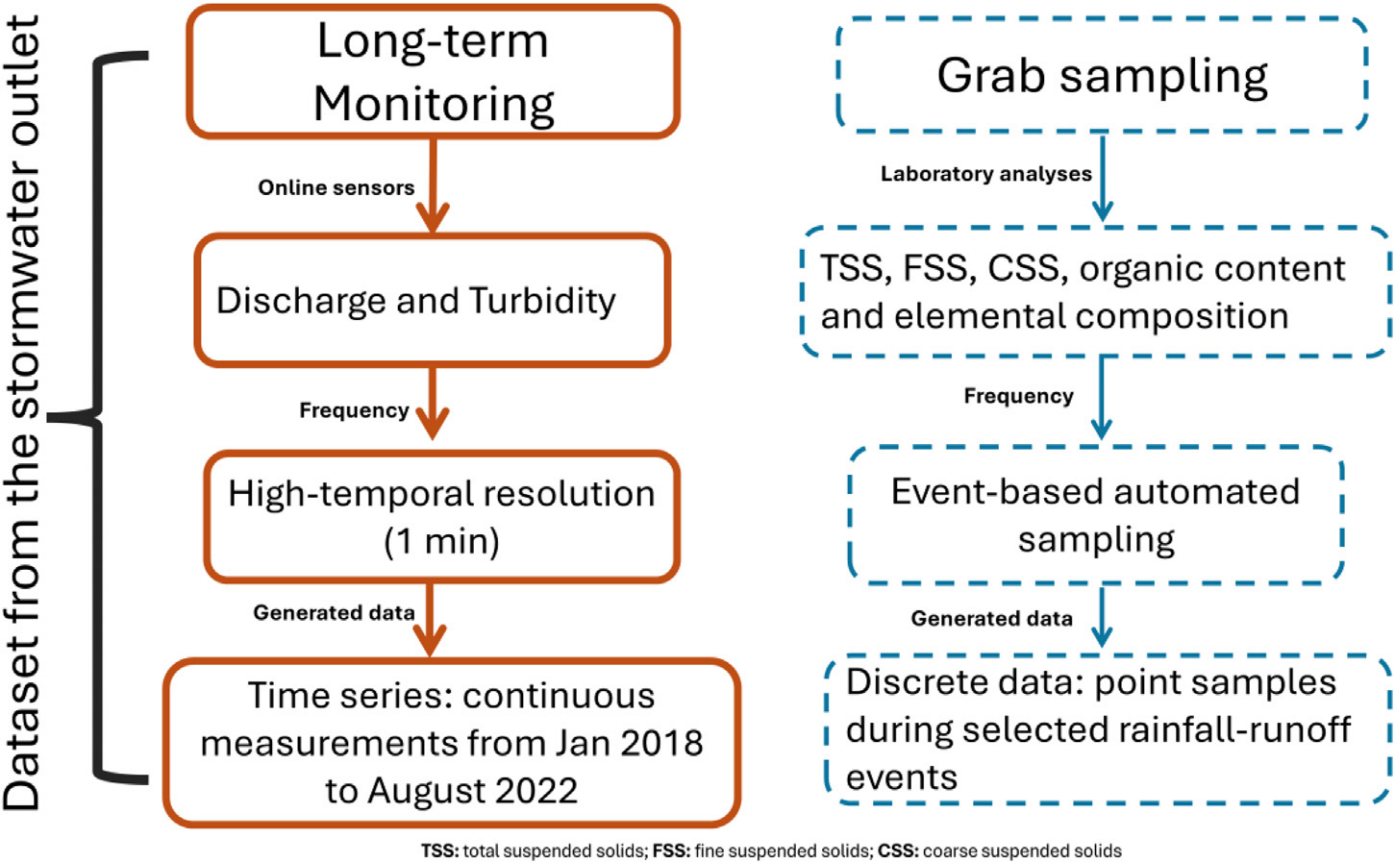
Workflow to generate the stormwater quality dataset using online monitoring and grab sampling.

The dataset was generated by combining onsite online monitoring at the outlet of the stormwater sewer network (SWO) and grab sampling of stormwater during rainfall events (Fig. 1).

### 4.1 Long-term and continuous monitoring at the SWO

The dataset comprises high-resolution turbidity and discharge data continuously measured from January 2018 to August 2022 in a monitoring station installed at the stormwater outlet (SWO) of an urban sub-catchment in Dresden, Germany (51.004014° N, 13.844630° E). This station is part of the monitoring network operated by the Chair for Urban Water Management of TU, Dresden [1,6,7].

The monitored urban sub-catchment (MS5) covers 23.6 ha (43 % impervious surfaces) and a separate sewer network drains it. The stormwater network (total length 2700 m) discharges to the nearby stream Lockwitzbach (mean flow 0.34 m^3^ s^−1^; discharge point: 51.004287° N, 13.8445545° E), which is a tributary of the Elbe River [1,6,7]. Rojas-Gómez et al. [1] show further details regarding the characteristics of the stormwater sewer network.

**Fig. 2:**
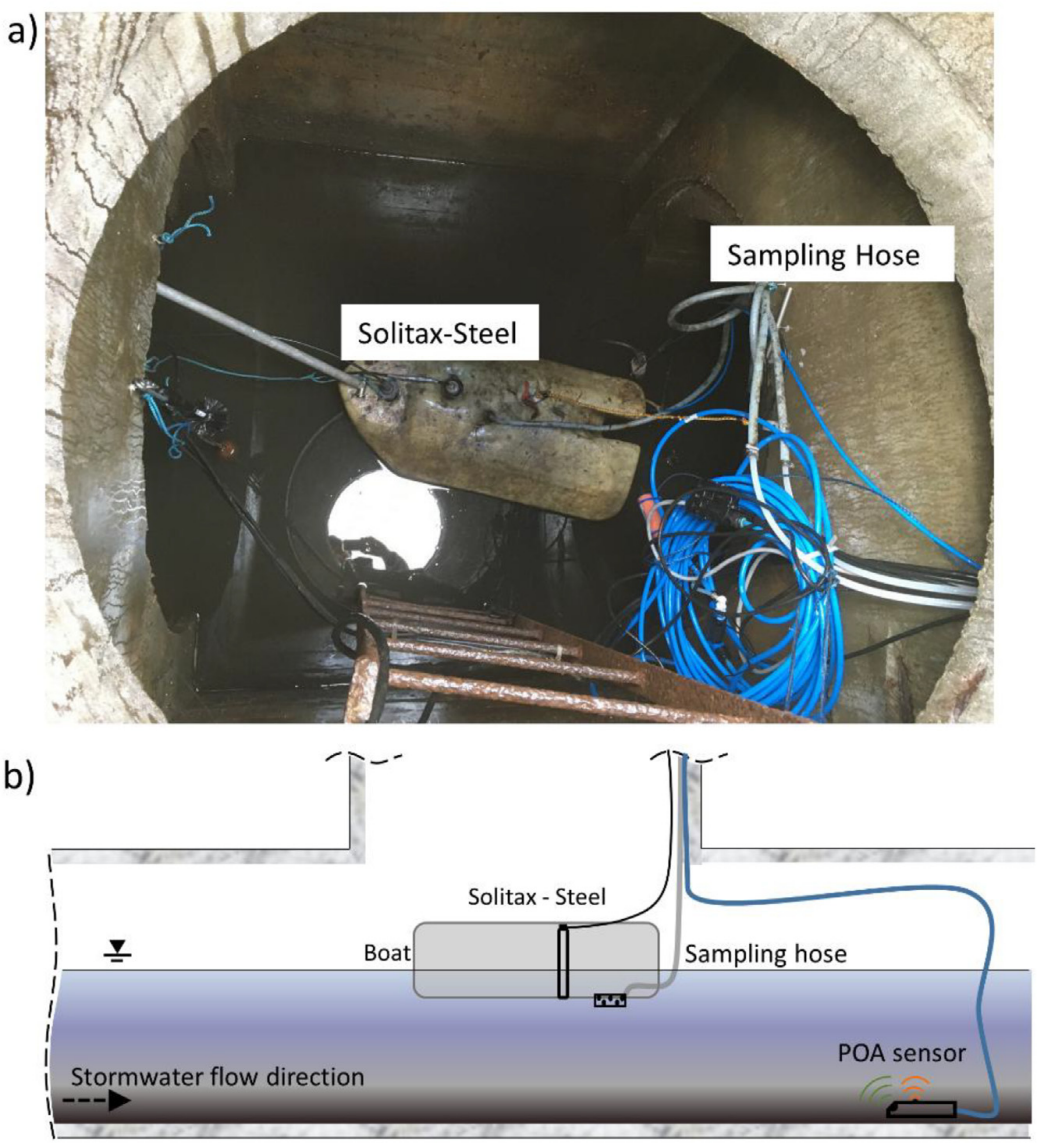
Sensors placed in the boat at the SWO for measuring turbidity and discharge (a) and the schematic representation of the installed sensors (b).

The online monitoring station measures turbidity at a 1 min time resolution using a Solitax-SC turbidity sensor (Hach, USA) housed in a steel case. Discharge is measured using a wedge sensor, which records velocity (via the cross-correlation method) and water level (pressure cell: POA-Sensor, NIVUS, Germany). The turbidity sensor is installed in a boat, located at the SWO (Fig. 2). This monitoring point within the sewer pipe is approximately 98 m upstream of the receiving stream. This last section of the sewer has a small longitudinal slope and its invert level is situated approximately at the same elevation as the receiving streambed. Therefore, this section of the sewer is permanently inundated with backwater from the stream, allowing the sensors to operate under constant wet conditions, limiting their exposure to dry periods and preventing the corrosion generated by alternating wet–dry conditions. No corrosion was observed during our monitoring period. Since groundwater infiltration was not relevant, groundwater–sewer interactions were beyond the scope of our study.

**Fig. 3:**
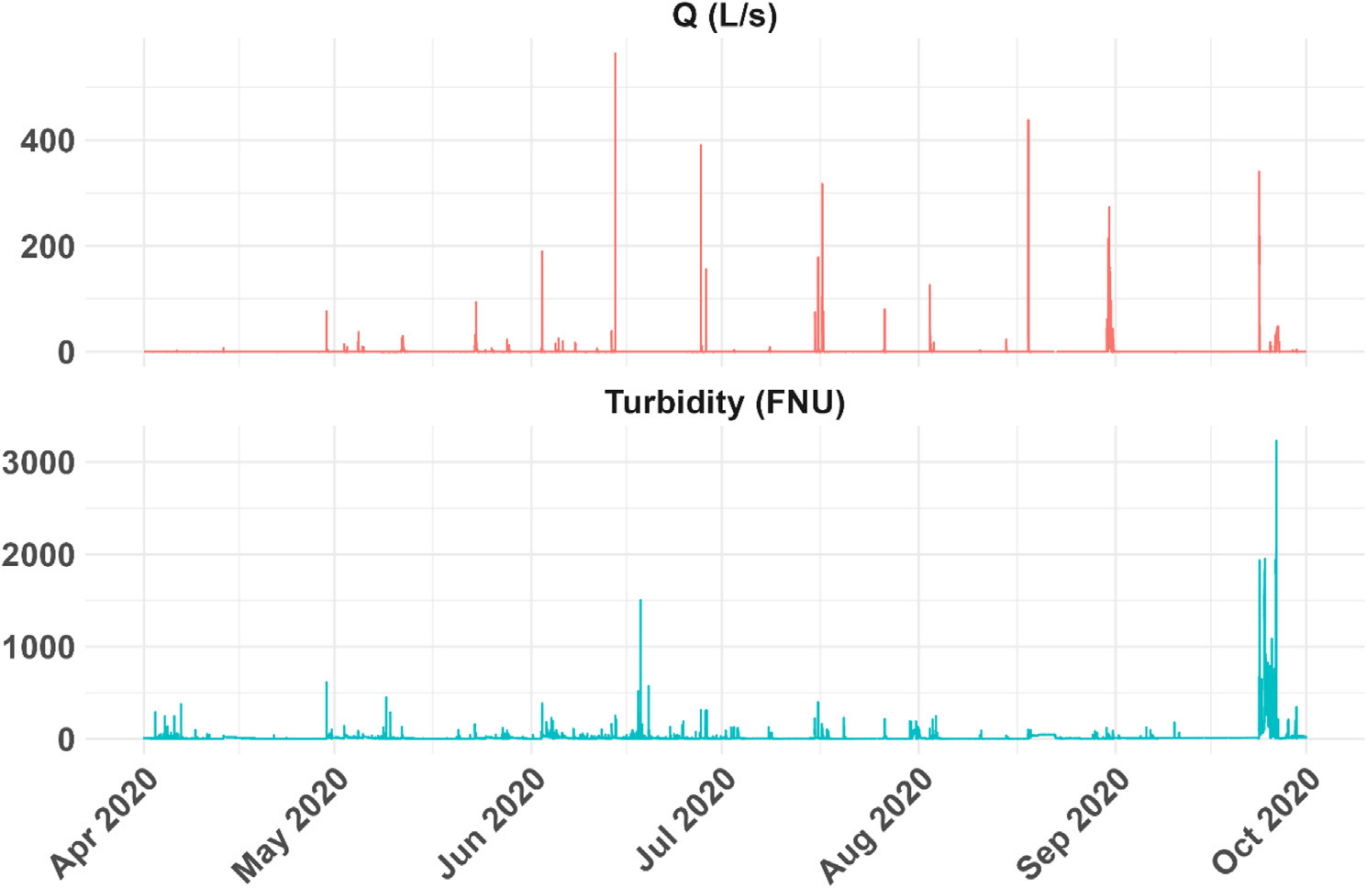
Example of continuous discharge (Q in L/s) and turbidity (FNU) raw data measured from April to October 2020.

The sensors were calibrated before their installation. Afterwards, the event-based grab samples were used to generate site-specific regression equations to relate turbidity and suspended solids for further calibration (see examples in Rojas-Gómez et al. [1]). Real-time data transfer facilitated the definition of maintenance frequency, requirements, data management, visualisation and quality control. Here, we present an example of the discharge and turbidity data from the Solitax-steel sensor (Fig. 3). Additionally, weekly maintenance of the monitoring station included cleaning the sensors, downloading data loggers, and checking their functionality. Further details on sensor cleaning dates, maintenance intervals, and quality control measures for the long-term online monitoring station MS5 are shown in the supplementary material (Table S1).

### 4.2 Sampling campaign and laboratory analysis

**Fig. 4:**
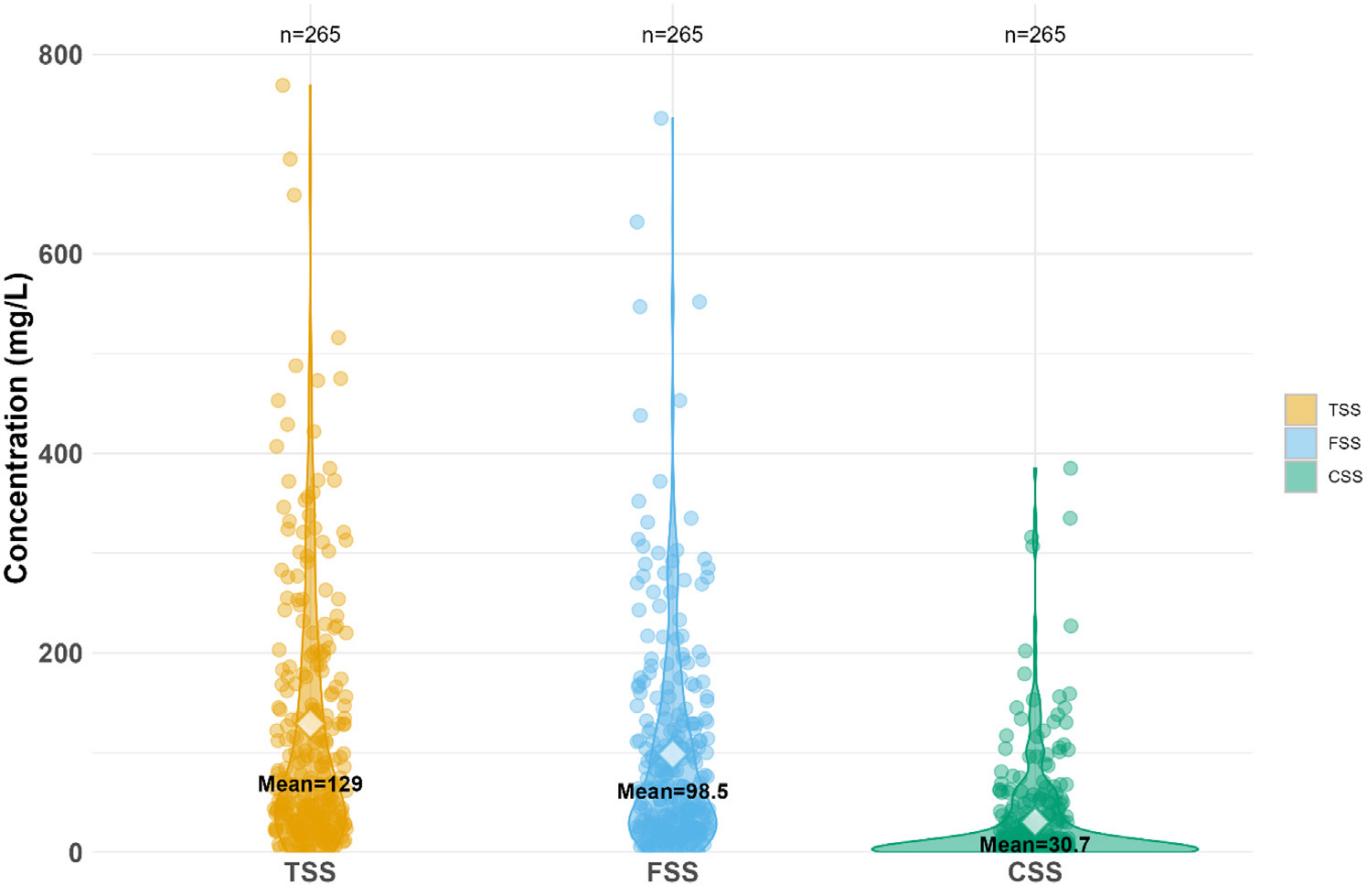
Distribution of total suspended solids (TSS), fine suspended solids (FSS) and coarse suspended solids (CSS) in the collected stormwater samples.

**Fig. 5:**
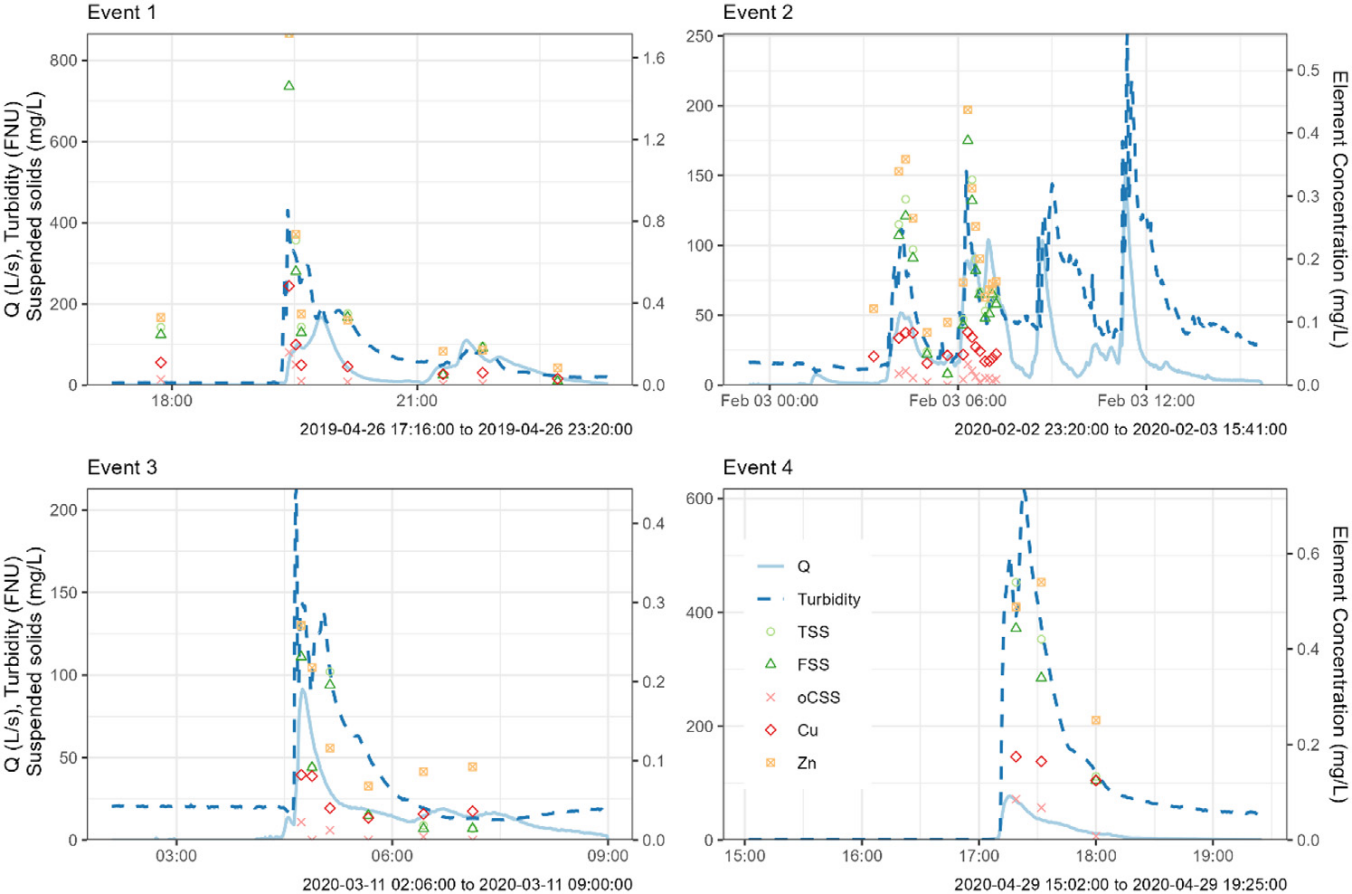
Four sampled runoff events containing data of discharge (Q), turbidity, total suspended solids (TSS), fine suspended solids (FSS), organic fraction of coarse suspended solids (oCSS), and elemental composition (e.g., Cu, Zn) in bulk sample. Modified from Rojas-Gómez et al. [1].

A sampling hose connected to an automatic sampler (MAXX GmbH) collects stormwater during rainfall events (Fig. 2). The sampling was carried out proportionally to volume, taking one sample every 30 m³, until all available bottles were filled (*n* = 24, volume per bottle: 1 L). The collected stormwater samples during rainfall events (September 2018 - 2021) were analysed for TSS, fine (<63 µm) and coarse (>63 µm) fractions, organic content, and elemental composition (Table 1). For the sample fractionation, a representative volume (200 mL) of each sample at a given time was wet sieved using stainless sieves (ø 50 mm, high 25 mm, test sieve DIN ISO 3310–1, VWR, Germany) with a mesh size of 63 µm. Subsequently, the fractionated sub-samples of fine suspended solids (FSS) and coarse suspended solids (CSS) were filtered separately using 0.45 µm cellulose nitrate membrane filters (Sartorius, Germany) following the method reported by Zhang et al. [8]. The soluble fraction corresponds to particles < 0.45 µm in accordance with DIN EN ISO 14,688–1. Then, the filters were evaporated at 105 °C until reaching constant weight to compute dry matter. The distribution of suspended solids is shown in Fig. 4. The organic matter content of the fine and coarse fraction of suspended solids was measured by the Loss on Ignition (LOI %) method (DIN EN 15,169, 2007) at 550 °C for 2 h [9]. For four selected storm events (32 stormwater samples) the concentration of elements (i.e., Al, B, Ba, Ca, Co, Cr, Cu, Fe, K, Li, Mg, Mn, Na, Ni, P, Pb, Sr and Zn) was determined by inductively coupled plasma optical emission spectrometry (ICP-OES, PerkinElmer, USA) in bulk samples (Fig. 5). The elemental composition was measured by drying the samples in an oven at 105 °C, homogenising, and digesting in a microwave with 5 mL H_2_O and 5 mL HNO_3_. The detection limit is shown in Table 2.

**Table 1:** Parameters measured at the stormwater outlet: Discharge (Q), turbidity, total suspended solids (TSS), fine suspended solids (FSS), coarse suspended solids (CSS), organic content of the fractions (oFSS and oCSS) and elements.

Variable	Frequency/number of samples	Data collection method
Q, Turbidity	1 min	Continuous monitoring
TSS, FSS and CSS	*n* = 265	Grab sampling during rainfall events
TSS, FSS, oFSS, CSS, oCSS	*n* = 157	Grab sampling during rainfall events
TSS, FSS, oFSS, CSS, oCSS, elements (Al, B, Ba, Ca, Co, Cr, Cu, Fe, K, Li, Mg, Mn, Na, Ni, P, Pb, Sr and Zn)	*n* = 32	Grab sampling during rainfall events

**Table 2:** Detection limit of the element concentration measured by the ICP-OES.

**Element**	**Al**	**B**	**Ba**	**Ca**	**Co**	**Cr**	**Cu**	**Fe**	**K**	**Li**	**Mg**	**Mn**	**Na**	**Ni**	**P**	**Pb**	**Sr**	**Zn**
Detection limit (mg L^−1^)	0.010	0.010	0.005	0.100	0.005	0.005	0.005	0.005	0.500	0.010	0.020	0.001	0.500	0.020	0.200	0.010	0.002	0.005

## Limitations

• Due to time and budget constraints, it was possible to sample only a limited number of rainfall events
• Our sampling strategy was focused on point samples, rather than composite samples per event, limiting the volume of water available for laboratory measurements. Grab samples collected during events had a maximum volume of 1 L for measuring suspended solids (TSS, CSS, and FSS) and their elemental composition. This volume was insufficient to measure all elements in all cases, and hence it was only possible to measure the elemental composition of four events. Hence, data on elemental composition are scarce compared with TSS data
• The reported element concentration was measured in the bulk sample (includes dissolved and particle-bound fraction)

## Ethics Statement

The authors confirm that they have read and follow the ethical requirements for publication in Data in Brief. Our current work does not involve human subjects, animal experiments, or any data collected from social media platforms.

## Funding

Open Access funding enabled and organized by Projekt DEAL. The Helmholtz International Research School TRACER (grant number HIRS-0017) funded the doctoral research of the first author K. L. Rojas-Gómez. This research was additionally funded by Helmholtz Water Science Network, Thematic Field 3: Urban Water Systems, and by the Deutsche Forschungsgemeinschaft (DFG, German Research Foundation); project Urban Resistom, grant number 460816351.

## CRediT authorship contribution statement

**Karen L. Rojas-Gómez:** Methodology, Software, Data curation, Investigation, Visualization, Writing – original draft. **Jakob Benisch:** Investigation, Data curation, Methodology, Writing – review & editing. **Björn Helm:** Methodology, Writing – review & editing. **Dietrich Borchardt:** Supervision, Funding acquisition, Project administration, Writing – review & editing. **Peter Krebs:** Supervision, Funding acquisition, Project administration, Writing – review & editing.

## Data Availability

OPARAMonitoring data to explore particle size distribution and elemental composition in a stormwater outlet from a German urban catchment (Reference data)

## Appendix Supplementary materials

Image, application 1
