# Supplementary material for: Monitoring data to explore particle size distribution and elemental composition in a stormwater outlet from a German urban catchment
Source: Data Brief. 2026 Feb 13;65:112581. doi: 10.1016/j.dib.2026.112581 (PMC12936563; doi:10.1016/j.dib.2026.112581)
Supplement: Supplementary file 1 [file mmc1.pdf]

Data in Brief

Supplementary material: Monitoring data to explore particle size distribution and elemental composition in a stormwater outlet from a German urban catchment

Karen L. Rojas-Gómez [a,b]*, Jakob Benisch [a] , Björn Helm [a], Dietrich Borchardt [b,c] and Peter Krebs [a]

[a] Institute of Urban Water Management, Technische Universität Dresden, 01069 Dresden, Germany
[b] Department of Aquatic Ecosystem Analysis and Management, Helmholtz-Centre for Environmental Research (UFZ), 39114 Magdeburg, Germany
[c] Institute of Hydrobiology, Technische Universität Dresden, 01069 Dresden, Germany

E-mail: *jakob.benisch@tu-dresden.de; bjoern.helm@tu-dresden.de; dietrich.borchardt@ufz.de; peter.krebs@tu-dresden.de*

* Corresponding author:

E-mail address: karen.rojas@tu-dresden.de

ORCID: K.L. R.-G.: 0000-0001-7097-7595; J. B.: 0000-0002-4782-6024; B. H.: 0000-0003-4963-7523; D. B.: 0000-0002-6074-2829

Electronic Supplementary Material

This document provides additional information on methods

Table S 1. Sensor cleaning dates and observations from the monitoring station MS5 (Stormwater sewer network)

Date	Cleaning Time	Observations/Comments	Solitax (Steel) Turbidity FNU		
			Before cleaning	After cleaning	Remarks
2017-12-05 13:00:00	13:00	installation of SC1000(Anise, 2TurbiditySensors) and Scan			
2017-12-13 12:10:00	12:05				
2001-12-20 14:20:00	14:17				
2018-01-04 13:00:00	13:07				
2018-01-10 11:30:00	11:32				
2018-01-16 10:20:00	10:29				
2018-01-24 11:15:00	11:22				
2018-01-31 12:15:00	12:17				
2018-02-08 12:04:00	12:17			4,38	
2018-02-16 15:25:00				5,4	
2018-02-22 14:12:00				4,41	
2018-03-01 12:38:00	12:43	Tests for Cond.Sensors		0,948	
2018-03-14 13:00:00	13:24	Event 2nd sample at 28.03.2018 23:28; 3rd sample 23:29 ; 4th sample 23:42; 5th bottle 23:43; 6th bottle midnight			
2018-03-29 16:03:00					
2018-04-11 08:10:00	08:12				
2018-04-20 10:29:00	10:51				
2018-04-25 08:50:00	11:13				
2018-04-27 10:10:00		Power cut			
2018-05-04 10:10:00	10:25				30,2
2018-05-11 16:20:00	16:23				
2018-05-18 11:43:00	12:02	12:24:00		6,38	6,38/20,7
2018-05-22 16:39:00	16:55			2,73	2,73/3,65
2018-05-29 14:40:00	14:38				
2018-05-30 09:52:00				2.76	
2018-06-05 12:55:00	13:00			9.82	
2018-06-13 12:25:00	12:40	Water level measurement failed during heavy rainfall, new zero line spectrolyser	4.85	20.5	
2018-06-20 13:38:00	13:50		2.99	6	
2018-06-27 12:30:00	12:32		6	5.9	
2018-07-05 11:20:00	11:30		4.42	9.82	
2018-07-12 13:26:00	14:05		12.2	14.7	
2018-07-18 10:19:00	10:38	very low water level, no measurement		9.6	
2018-07-26 11:02:00	11:10	Installation of new discharge sensor (cs2 removed, poa installed)	18.7	47.8	
2018-08-02 12:54:00	13:01	scan dry, anise dry and redox dry, turb also dry. Still questionable if new wedge sensor works, no proper velocities recorded!	6.59	11.7	
2018-08-09 11:13:00	11:29		12.7	45.6	
2018-08-15 12:26:00	12:30	small nitrate value measured, sample is going to be analyzed in the lab for further info	28.3	71.5	
2018-08-22 13:44:00	13:55	cooling function for sampler in mastervolt available	2.4	5.45	
2018-09-05 16:07:00	16:09		2.24		
2018-09-11 11:33:00	11:45	scan sensor was blocked (previous peak!)	4	49	
2018-09-20 12:34:00		sewer dry, sensors were measuring in air	10.7		plastik working, steel not working
2018-09-23 12:30:00	12:45				
2018-10-03 11:44:00	11:50	no water in channel, scan dry,	7.09	9.88	steel dry
2018-10-12 12:04:00	12:10	powercut, creekwater in the channel	10.2	9.71	

2018-10-19 12:29:00	12:27	no water in channel, scan dry, anise still workin	0.26		
2018-10-30 11:13:00	11:20	water again!	8.2	9.8	
2018-11-05 13:55:00	14:00		5.37	6.89	
2018-12-05 11:18:00	11:39	powerlosss due to works in the garden<br>next to the station	50.4	50.6	
2018-12-13 12:23:00	12:27	powercut du to unknown reason, new zero spectrolyser, new sampler 12:52/53 measurement	22.1	20.7	
2018-12-19 12:08:00	12:11	much water, lots of sediment. Sampler: warning of „contamination“ took no samples	5.57	9.6	
2019-01-10 14:45:00	15:05		22.2	19.7	
2019-01-17 15:31:00	15:37		16.3		
2019-01-22 16:21:00			4.31	9.34	
2019-02-01 13:55:00	13:59	sampler installed	12.7	12.8	
2019-02-06 14:58:00	15:05	high salinity	13.5	15	
2019-02-20 15:20:00	15:25			34	
2019-02-28 14:28:00	14:33	small Layering (340-410)	7.44	4.41	
2019-03-06 11:18:00	11:28	almost no Layering, 176-180	55	60.9	
2019-03-12 12:34:00	12:39	scan zero, No Layering	21.5	28.5	
2019-03-21 16:10:00	16:14	small Layering 295-395	5.69	6.07	
2019-03-29 10:51:00	10:55:00	Layering 80-252	46.6	47	
2019-04-03 09:20:00	09:28	small Layering (240-340)	8.78	12	
2019-04-12 13:10:00	13:19	Minimal Layering (444-464)	1.45	88	
2019-04-17 12:42:00	12:46	small Layering (280-350)	12.7	16	
2019-04-26 11:15:00	11:20	installation of old cartridge, too less water for Layering	4.49	9.7	
2019-04-29 09:50:00	09:50	Event		59.6	
2019-05-08 11:48:00	11:53	small Layering 80-280)	4.16	4.64	
2019-05-20 13:11:00	13:20	No Layering – abfluss	8.8	35	
2019-05-27 12:13:00	12:20	Minimal Layering (202-212)	2.25	5.5	
2019-06-04 14:48:00	14:52	No Layering	2.1	3.5	
2019-06-12 12:28:00	12:42	Minimal Layering (99-111)	11.3	10	
2019-06-24 12:51:00	12:58	mall Layering (840-885)	44.9	42.4	
2019-07-08 12:03:00	12:07		7.36	11.4	
2019-07-22 14:42:00	14:47	Almost no water, powercut, cable protection was deteriorated	64.4	37	Steel out of water
2019-07-29 12:48:00	12:52	Too little water for layering	5.86	124	out of water
2019-08-08 13:46:00	14:04		8.1	15.4	
2019-08-16 11:23:00	11:25	Too little water for layering	5.78	40.8	
2019-08-27 09:52:00	09:55	Too little water for layering	8.2	21.5	
2019-09-03 14:54:00	15:02	Too little water for layering	3.27	13.1	
2019-09-20 09:54:00	09:57	Too little water for layering	28.6	5.1	
2019-09-26 08:51:00	08:56	Too little water for layering	1.8	5.8	
2019-10-02 12:49:00	12:53	No Layering	22	24	
2019-10-17 13:53:00	13:54	smelly sludge, fouling	30	56	steel dry
2019-10-22 10:12:00	10:17	scan no zero line- bidest spectra after cleaning was fine, bidest before acid: 10:22-10:24, bidest after acid: 10:29-10:31<br>No Layering	12	33	
2019-11-01 12:02:00	12:06	No Layering	3.8	6.7	
2019-11-08 12:09:00	12:13	installation of two samplers<br>No Layering	9.5	5.9	
2019-11-21 13:23:00	13:30	No Layering	7.45	17.6	
2019-12-04 10:32:00	10:37	No Layering	4.8		

2019-12-13 08:45:00		No Layering	10	10.8	
2019-12-18 11:26:00	11:32	No Layering	14.4	16.6	
2020-01-02 11:58:00	12:02	installation of hobo conductivity. No Layering	2.81	1.83	
2020-01-09 11:30:00	11:40	small event	60	42	
2020-01-15 11:51:00	12:01	almost no water left, change of sampling hoses, both are now at the boat, change of sampling volume	8.53	23.7	
2020-01-22 11:23:00	12:33	Slight Layering (309-490)	8.7	11.4	
2020-01-31 12:26:00	12:28		11.3	41	
2020-02-06 12:09:00	12:20	Slight Layering (130-260)	7.73		
2020-02-11 10:51:00	11:02	No Layering	22.8	24.1	
2020-02-18 14:11:00	14:32	No Layering	10.5	18.7	
2020-02-26 10:28:00	10:33	sampler removed, No Layering	61	59.6	
2020-03-05 12:18:00	12:22	sampler removed. Slight Layering (290-390)	10	10.2	
2020-03-26 11:33:00	11:36	Almost no Layering (311-322)	3.16	5	
2020-04-02 11:05:00	11:11	Layering 190-312	5.4	5.77	
2020-04-09 10:58:00	11:04	No Layering	1.26	7.2	
2020-04-16 11:14:00	11:20	Slight Layering (270- 190)	11	12	
2020-04-23 10:00:00	10:05	exchange of compressor. No Layering, (460-467)	1	2.9	
2020-04-30 13:36:00	13:48	No Layering	15.6	22.6	
2020-05-07 10:53:00	11:00	No Layering	7.7	9.5	
2020-05-13 10:52:00		No Layering	3.8	5.65	
2020-05-20 11:09:00	11:18	No Layering	1.22	9.23	
2020-05-28 09:43:00	09:43	compressor melted. Layering (86 466)	7.38	7.34	
2020-06-04 09:33:00	09:40	still no compressor. Slight Layering(77-84)	24.8	45	
2020-06-25 10:56:00		Nivus sensor with some sand	4.06	16.9	
2020-06-28 09:40:00		Cleaning of the sample hose from the boat			
2020-06-29 13:40:00		nivus added			
2020-07-02 10:37:00	10:42	sand in the channel. Sand removed, sampling tubes reinstalled no Layering	3.5	18	
2020-07-08 12:27:00	12:35	small event. No Layering	20.3	11	
2020-07-16 10:40:00	10:45	The boat landed on the shore of sewer. no Layering after event	9.13	7.3	boat landed on the shore of sewer
2020-07-23 09:47:00	09:52	dried out. no water in channel, scan dry, anise still working	1.06	3.91	dried out
2020-07-30 12:00:00		little water	3	3	dried out
2020-08-06 09:39:00	09:42	little water	4	4	dried out
2020-08-13 11:29:00	11:34	no water in channel, scan dry, anise still working	3.9		dried out
2020-08-26 10:14:00	10:17	no water in channel, scan dry, anise still working	6.48	3.7	dried out
2020-09-01 10:45:00	10:47	Discharge sensor blocked by debris water after event, slight Layering (84-117)	11.7	17.8	
2020-09-07 13:15:00	13:18	No Layering	6.9		
2020-09-07 13:15:00					
2020-09-23 10:10:00	10:16	powercut, no water	9.3		dried out
2020-09-24 09:49:00	09:40				
2020-09-29 10:40:00	10:43	Event, sediments in channel	13	8.67	
2020-10-05 10:40:00					
2020-10-13 10:25:00	10:30	Little water	23.2	89	
2020-10-28 11:25:00	11:30	Powercut. Layering (108, 180)	9.98	48	
2020-11-11 13:17:00	13:20	Slightly Layering (404-411)	40	13	
2020-11-24 13:18:00	13:20	Slightly Layering (230-290)	7.66	50	

2020-11-30 11:36:00		pc crashed			
2020-12-15 11:46:00	11:48			856	
2021-01-08 10:54:00	10:58	Slightly Layering (245-265)	18	15.8	
2021-02-03 14:13:00	14:15	Slightly Layering 421-490	3.8	6.21	
2021-02-16 13:23:00	13:30	No Layering	4.1	4.2	
2021-02-26 10:18:00	10:25	Slightly Layering (408-422)	3.1	14	
2021-03-05 13:36:00		Layering (193-350)			
2021-03-12 11:20:00	11:40	No Layering	64.2	77	
2021-03-19 12:10:00	12:12	No Layering	5.5	5.8	
2021-03-24 14:19:00	14:32	fine sediment in sewer. Slightly Layering (330-430)	7.5	45	
2021-04-01 11:23:00	11:26	No Layering (426)	1.36	6.38	
2021-04-09 12:03:00	12:08	sampler fuse needs to be replaced. Slightly Layering (100-340)	13	15.4	
2021-04-20 10:58:00	11:07	Slightly Layering (64-80)	19.7	21.2	
2021-05-10 10:20:00	10:25	Slightly Layering (140-170)	30	25	
2021-05-15 19:14:00	19:16	power cut			
2021-05-19 11:13:00	11:30	No Layering	60	44.6	
2021-05-31 13:35:00	13:40				
2021-06-01 12:12:00	12:30	removal of scan sensor was drifting too much. small Layering (290-310)	7.25	7.19	
2021-06-16 10:53:00	12:09	installation of another spectrolyser	5.93	95.2	
2021-06-24 12:13:00		sampler error. No layering	9.5	10.6	
2021-06-29 13:59:00	14:04	removal of ise from water, sampler could be broken. Small Layering (155-280)	6.2	19	
2021-07-01 13:00:00	13:09				
2021-07-06 12:57:00	13:00	installation of new LTE stick<br>small Layering (350-320)	0.5	2.73	
2021-07-15 13:12:00	13:43	boat was lifted and is not in water anymore. Slightly Layering (100-140)	0.27	5.8	
2021-07-22 11:34:00	12:03	sampler broken, relocation of turbidity sensor<br>Layering (480-610)	3.2	5.4	
2021-08-05 13:01:00	13:05	sampler reinstalled. No Layering	23		
2021-08-10 13:01:00	13:04	power cut. No Layering	2	2.5	
2021-08-19 15:30:00		power cut			
2021-09-08 13:50:00	13:44				
2021-09-16 10:31:00		power cut. No Layering	14	14	
2021-09-23 12:49:00	13:00	small Layering (145-312)	3.17	3.22	
2021-09-30 08:57:00	09:01	small schichtugn (100-140)	3.3	4.18	
2021-10-12 14:40:00	14:47	No Layering	31	30	
2021-10-21 13:00:00		power cut, socket was loose			
2021-11-02 12:00:00	13:45	Installation of level gauge nivus	7.3	10	
2021-11-04 12:24:00	12:32	power cut			
2021-11-12 16:52:00		adjustments of nivus sensor	1		
2021-11-24 09:50:00	10:03	Layering (219-455)	3.06	5.6	
2021-12-01 11:34:00	11:40	No Layering	126	47	
2022-01-06 13:45:00	14:18	No Layering	13	13	
2022-01-12 14:59:00	15:00	power cut. No Layering	8.66	14	
2022-01-21 14:10:00	14:18	Small layering (360-350)	16.7	30	
2022-02-03 15:36:00	15:48	no Layering (390 -393)	17.6	25.8	
2022-02-07 14:27:00		power cut			
2022-02-09 11:29:00	11:32	change of compressor	18.1	33.5	
2022-02-17 14:24:00	14:08	sensor cleaning			
2022-02-22 10:43:00	10:50	modem error, installation of new sampler to exchange top sampler,<br>Layering (90-350)	16	14	

2022-03-08 11:04:00	11:14	modem error, No Layering	2	9.5	
2022-03-16 12:08:00	12:17	Layering (66,72,5)	19	23	
2022-03-22 14:48:00	14:54	No Layering	1.36	7	
2022-03-30 12:09:00	12:30	No Layering	8.89	14.4	
2022-04-05 11:02:00	11:20	No Layering	31	30	
2022-04-11 16:48:00		turn around of the acoustic turbidity sensor			
2022-04-22 10:34:00		No Layering	1.2	28	
2022-05-05 11:00:00	11:22	Little layering (499-516)	2.6	13.4	
2022-05-23 15:40:00	15:42				
2022-05-25 15:20:00	15:15	No Layering		29	
2022-06-01 09:55:00	10:04	removal of sampler	29.3	43	
2022-06-09 13:15:00	13:17	No Layering	22.7	28.4	
2022-06-15 11:45:00	11:48		13	44	
2022-06-22 11:21:00	11:28		2	7.9	
2022-06-30 10:18:00		removal of acustic turbidity sensor			
2022-07-13 09:24:00	09:27	removal of bottom turbidity sensor (solitax)	2	13.9	removal of bottom solitax
2022-07-19 11:30:00	11:35		1.7	6.6	
2022-07-27 12:28:00	12:36		15.7	22.9	
2022-08-03 10:19:00	10:16	No Layering	15.6	49	
2022-08-20 10:53:00	10:58	event	2889	17.9	
2022-08-29 12:50:00	12:55	Layering (90,374)	2411	8.64	
